# Combined clinical and ultrasound criteria could accurately predict the Y chromosome in primary amenorrhea patient

**DOI:** 10.1038/s41598-023-49570-8

**Published:** 2023-12-13

**Authors:** Kanadi Sumapraja, Niken Pudji Pangastuti, Muhammad Ikhsan, Achmad Kemal Harzif, Mila Maidarti

**Affiliations:** 1 Reproductive Immunoendocrinology Division, Department of Obstetrics and Gynecology, Faculty of Medicine, Universitas Indonesia - Dr. Cipto Mangunkusumo General Hospital, Jalan Pangeran Diponegoro No. 71, Jakarta, 10320 Indonesia; 2 Yasmin IVF Clinic, Dr. Cipto Mangunkusumo General Hospital, Jakarta, Indonesia; 3 Human Reproduction, Infertility, and Family Planning Cluster, Faculty of Medicine, Indonesia Reproductive Medicine Research and Training Center, Universitas Indonesia, Jakarta, 10430 Indonesia

**Keywords:** Endocrine reproductive disorders, Genetics research

## Abstract

This study aimed to assess the combined clinical and ultrasound criteria as a diagnostic tool for screening the Y chromosome related to primary amenorrhea. This cross-sectional study involving 59 subjects was taken from medical records at the Reproductive Immunoendocrinology Polyclinic of Cipto Mangunkusumo General Hospital, Jakarta, Indonesia. The medical records of subjects were then cross-checked with karyotyping analysis results. Sensitivity, specificity, and predictive values were analyzed to assess the criteria. Two subjects were presented with a Y chromosome, and one without a Y chromosome was misclassified into another group. After analysis, we found that combined clinical and ultrasound criteria could predict the Y chromosome related to primary amenorrhea with 95.9% accuracy, with sensitivity and specificity of 80% and 97.96%, respectively. Combined clinical and ultrasound criteria (introduced as Kanadi Sumapraja Criteria) could be used as a diagnostic tool for screening a Y chromosome related to primary amenorrhea.

## Introduction

Primary amenorrhea is a symptom characterized by the absence of menstruation in 14-year-old women without secondary sexual development or 16-year-old women with secondary sexual development^1^. The incidence of primary amenorrhea is up to 1–3% of reproductive women^2^. Several studies showed that gonadal dysgenesis, Mullerian duct agenesis, and central amenorrhea are the most common aetiology of primary amenorrhea^3^.

Chromosomal analysis in women with primary amenorrhea is necessary to diagnose and appropriately manage the patient accurately. Several studies showed that chromosomal abnormalities may contribute up to 16–50% of the aetiological factor of primary amenorrhea^4^. One of the conditions with primary amenorrhea may be related to the disorder of sexual development (DSD). In DSD, excess androgen exposure and Y chromosome involvement might exacerbate the condition with the risk of germinal cell neoplasia in undifferentiated testis (gonadal dysgenesis). Hence it needs the multidisciplinary approach as a complex case for early diagnosis and management of DSD, including the karyotyping analysis as an essential assessment for diagnosis^5^.

Unfortunately, in Indonesia, the accessibility of chromosomal analysis is confined to specific centres. This limitation poses a considerable challenge for physicians in accurately determining the underlying causes of primary amenorrhea. The existing diagnostic framework for primary amenorrhea lacks the capacity to detect the presence of the Y chromosome in such cases. Consequently, developing an assessment tool utilizing clinical parameters like medical history, physical examination, and ultrasound is paramount. This tool would enable the identification of Y chromosome involvement in patients with primary amenorrhea. The idea of developing this combined clinical and ultrasound criteria was to develop an easier prediction of the Y chromosome for use where karyotyping facility access is unavailable, especially in rural areas of Indonesia.

In 2016, an experienced clinician in DSD created combined clinical and ultrasound criteria. These criteria were designed to categorize cases of primary amenorrhea by considering several variables, including Tanner stage indicators (breast, axillary, and pubic hair development), external genitalia phenotype, and the presence of a uterus as observed through ultrasound. This classification system could predict the presence of the Y chromosome in individuals with primary amenorrhea. Subsequently, we have refined and expanded upon the original criteria and called it Kanadi Sumapraja criteria to encompass the full spectrum of phenotypic variations that could potentially arise. This enhanced criteria set is expected to offer valuable assistance in diagnosing and effectively managing patients experiencing primary amenorrhea with Y chromosome involvement.

## Methods

This was a diagnostic study with a cross-sectional approach using secondary data from medical records derived from the Reproductive Immunoendocrinology Clinic in Cipto Mangunkusumo National Referral Hospital, Jakarta, Indonesia. This study has been approved by the Ethical Committee of the Faculty of Medicine Universitas Indonesia with ethics number KET-860/UN2.F1/ETIK/PPM.00.02/2023. Informed consent was obtained from all participants or their legal guardians. This study has been performed in accordance with the Declaration of Helsinki and the regulations of the ethics committee of the Faculty of Medicine Universitas Indonesia.

The inclusion criteria of the subjects were primary amenorrhea patient; did not have a history of hormonal therapy; complete physical examination recorded in the medical record (including breast development examination and pubic hair development, external genitalia); ultrasound examination; and karyotyping analysis. Clinical examinations, including Tanner staging and ultrasound examination, were performed by the experienced clinician in the reproductive endocrinology clinic by the same clinician as the independent investigator.

Subject data included age, marital status, body mass index, Tanner stage, external genitalia, ultrasound examination, karyotyping analysis, and diagnosis. All the karyotyping analysis was performed in the Biology Laboratory of the Faculty of Medicine University of Indonesia.

Then, every subject was classified based on Kanadi Sumapraja criteria. In this criteria, there were five categories based on characteristics from clinical features and absence of uterus from ultrasound examination. Clinical features defined as breast and pubic hair development by Tanner stage (M1–M5 and P1–P5). The Kanadi Sumapraja table and its interpretation can be seen in Tables 1 and 2 below.

**Table 1:**
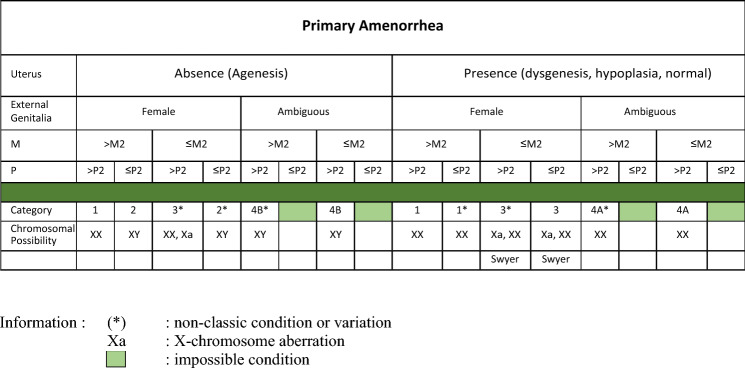
Combined clinical and ultrasound criteria or Kanadi Sumapraja criteria.

**Table 2:** Interpretation of Kanadi Sumapraja criteria.

Category	Description	Possible diagnosis	Chromosomal possibility
1	Abnormality in internal genitalia organ and reproductive tract	Mullerian duct dysgenesis, endometrial defect, or outflow tract anomaly	46 XX
2	Absence of internal genitalia organ, non-functioning androgen	Complete defect of synthesis or complete androgen sensitivity syndrome (CAIS)	46 XY
3	Defect of the hypothalamic-pituitary-ovary axis (HPO)	Central amenorrhea	46 XX, 45 XO,
		Primary ovarian insufficiency (POI), Turner syndrome, Swyer syndrome	X-chromosome aberration, 46 XY
4 A	Female too much androgen	Polycystic ovarian syndrome (PCOS), congenital adrenal hyperplasia (CAH)	46 XX
4 B	Male too little androgen	Partial defect of synthesis or partial androgen insensitivity syndrome (PAIS)	46 XY

In classifying using Kanadi Sumapraja criteria, there are several assessment steps. The initial step entails evaluating the presence of the uterus, followed by a comprehensive assessment of the external genitalia. Subsequently, the examination proceeds to the evaluation of breast development and the development of pubic hair.

It's important to note that while most criteria within this classification framework can be determined through clinical evaluation, distinguishing Swyer syndrome from other instances falling under class 3 criteria requires karyotyping analysis. Karyotyping is the definitive method to identify Swyer syndrome, characterised by a 46, XY karyotype despite female phenotype. This emphasizes the significance of karyotyping as a crucial diagnostic tool in cases that involve potential Y chromosome involvement.

Data analysis was presented in descriptive and analytic using SPSS statistical analysis software. Data were presented in a 2 × 2 diagnostic table. The accuracy, sensitivity, specificity, and predictive values were presented. The classifying diagnostic table is presented in Table 3 below.

**Table 3:** Diagnostic test table.

Reference index		Karyotype		Total
		Presence of Y-Chromosome	Absence of Y-Chromosome	
Kanadi Sumapraja Criteria	2, 4B	a	b	a + b
	1, 3, 4A	c	d	c + d
Total		a + c	b + d	N

## Results

Out of the 93 primary amenorrhea subjects at the clinic, 59 received karyotyping analysis. Their ages ranged from 14 to 37, with a median of 22 years. Notably, 30.5% were in the 17–20 age group, while only 11.9% were 16 or younger. Additionally, most were unmarried (80.65%) and primarily referred cases (96.7%). The details characteristics of subjects can be seen in Table 4.

**Table 4:** Subjects’ demographic characteristics.

Demographic characteristics	Total (n = 59)	Percentage (%)
A. Age group (years)		
16 or less	7	11.9
17–20	18	30.5
21–25	16	27.1
26–30	16	27.1
31 or more	2	3.4
Median 22,00 years (14–37)		
B. Marital status		
Married	7	11.9
Unmarried	52	88.1
C. Referral case		
Referral case	57	96.6
Came by herself	2	3.4

Most primary amenorrhea subjects exhibited a height range of 141–160 cm, accounting for 49.1%. Roughly 22.1% of subjects had a height of 140 cm or less, while 28.8% stood taller than 160 cm. Regarding breast development, a significant proportion, specifically 88.2%, were at Tanner stages M1 and M2. Merely 11.8% of subjects were at the M3 stage. Regarding pubic hair development, an equivalent proportion, 88.2%, were at stages P1 and P2. Notably, only two subjects (3.4%) demonstrated normal uterus development. However, uterine agenesis was observed in 10 subjects (16.9%). Furthermore, a small percentage of subjects, about 6.8%, exhibited ambiguous genitalia. Table 5 provides a comprehensive overview of the medical characteristics of the subjects.

**Table 5:** Subjects’ medical characteristics.

Medical characteristics	Total (n = 59)	Percentage (%)
A. Body Height		
121–130 cm	4	6.8
131–140 cm	9	15.3
141–150 cm	12	20.3
151–160 cm	17	28.8
161–170 cm	16	27.1
> 170 cm	1	1.7
Median 155 cm (122–175)		
B. Body Mass Index		
< 18 (underweight)	20	33.9
18–24 (normoweight)	28	47.5
> 24 (overweight)	11	18.6
Median 20 (12–35)		
C. Breast Development (Tanner Stage)		
M1	46	78
M2	6	10.2
M3	3	5.1
M4	2	3.4
M5	2	3.4
D. Pubic Hair Development (Tanner Stage)		
P1	43	72.9
P2	9	15.3
P3	1	1.7
P4	4	6.8
P5	2	3.4
E. Uterus Development (Ultrasound)		
Normal	2	3.4
Hypoplasia	43	72.9
Dysgenesis	4	6.8
Agenesis	10	16.9
F. External Genitalia Phenotype		
Female Phenotype	55	93.2
Ambiguous	4	6.8

Among the 59 subjects who underwent chromosomal analysis, 29 (49.2%) displayed chromosomal abnormalities. Notably, 10 cases (16.95%) exhibited the presence of the Y-chromosome, appearing as mosaicism or 46 XY. The diagnosis of primary amenorrhea was established through a comprehensive assessment encompassing medical history, physical examination, ultrasound, laboratory findings, and karyotyping analysis. The prevalent diagnosis was gonadal dysgenesis (57.6%), encompassing conditions such as Turner syndrome, Primary Ovarian Insufficiency (POI), and Swyer syndrome. Central amenorrhea followed at 18.6%, while androgen insensitivity syndrome accounted for 13.6% of cases.

Table 6 provides an overview of the underlying causes of primary amenorrhea in subjects.

**Table 6:** Aetiology of primary amenorrhea on subjects.

Amenorrhea Etiology	Total (n = 59)	Percentage (%)
A. Karyotyping Analysis Result		
46 XX	30	50.8
45 XO	5	8.5
Mosaicism	14	23.7
Mosaicism with a Y chromosome	1	1.7
46 XY	9	15.3
B. Diagnosis		
Turner Syndrome (including mosaicism)	16	27.1
POI	16	27.1
Central Amenorrhea	11	18.6
Androgen Insensitivity Syndrome	8	13.6
Mullerian Duct Anomaly	3	5.1
Delayed Puberty	2	3.4
Swyer Syndrome (including mosaicism)	2	3.4
Congenital Adrenal Hyperplasia (CAH)	1	1.7

We performed diagnostic test analysis on subjects classified according to Kanadi Sumapraja criteria to karyotyping analysis result as the reference standard (Table 7). The accuracy of Kanadi Sumapraja criteria in screening Y chromosome presence was 94.92%. The sensitivity and specificity of this criteria were 80% (49.02–94.33%; [95%-CI]); 97.96% (89.31–99.64%; [95%-CI]), respectively. The positive predictive value of this study was 88.89% (56.5–98.01%; [95%-CI]), and the negative predictive value of 96% (86.54–98.9%; [95%-CI]). The positive likelihood ratio was 39.2 (5.5–279.55; [95%-CI]), and the negative likelihood ratio was 0.2 (0.06–0.71; [95%-CI]).

**Table 7:** Diagnostic test of Kanadi Sumapraja criteria and chromosomal analysis result.

		Chromosomal analysis		Total
		Y related	Non-Y Related	
Modified Kanadi Sumapraja Criteria	Y Related	8	1	9
	Non-Y Related	2	48	50
Total		10	49	59

## Discussion

In this study, 49.2% of subjects had a chromosomal abnormality, varied in structural abnormality or aneuploidy, that might cause the primary amenorrhea. This study is relevant to previous studies which supported that chromosomal abnormality is common in primary amenorrhea with various percentages, 24.5% in Hongkong^6^; 27.8% in India^4^; 31% in Malaysia^7^; 34% in Sri Lanka^8^; 41.7% in Mexico^2^. Due to the high prevalence of chromosomal abnormality in primary amenorrhea patients, the chromosomal analysis ideally should be performed on all the women with primary amenorrhea.

The most common three diagnoses of primary amenorrhea in this study were Turner syndrome (27.1%), POI (27.1%), and central amenorrhea (11%). However, this finding differed slightly from the previous study, which showed that the most common primary amenorrhea diagnoses were gonadal dysgenesis, Mullerian duct agenesis, and central amenorrhea^9^.

We found that the subjects’ characters mostly had no development of pubic hair and breasts. Hence the subjects were categorized into category 3. Pubic hair development was influenced by the androgen hormone derived from the adrenal. Hypothalamus–pituitary–adrenal axis is not relatable with the hypothalamus-pituitary-ovarian axis^1^. Pubarche delay development in category three subjects was commonly followed by short stature, indicating the lack of growth spurt. Growth hormones influence the growth spurt. The bond between growth hormone with its specific receptors will stimulate the synthesis and secretion of insulin-like growth factor-1 (IGF-1) that will augment the effect of FSH and LH in ovaries. Moreover, it also will affect the ACTH in adrenal steroidogenesis and thyroid response to thyroid-stimulating hormone (TSH). Thus, the growth spurt delay will impact adrenal and ovary steroidogenesis, delaying the development of secondary sexual characteristics^1^.

Subjects with partial or complete androgen insensitivity commonly had a non-classical appearance. There was no breast development in two subjects classified in category 2 (M1). However, in the other subject that was classified into category 4B, there was significant breast development (M4). In 46, XY subjects with androgen insensitivity, breast development variety results from the absence or presence of peripheral aromatization into estrogen or lack of estrogen receptor sensitivity in the individual with 46, XY. Besides the ovary, estradiol can be produced from peripheral subcutaneous fat and skin. Even though the quantity of estrogen produced by adipocytes or fibroblasts is scant, the continuous production of estrogen will contribute to circulating estrogen, especially in obese subjects^10^.

These criteria unpredicted two subjects with Y chromosomes. The case included one subject with Swyer syndrome and one with DSD mosaicism 46, XO/46, XY. They had the same female phenotype with gonadal dysgenesis. Patients with Swyer syndrome typically have female phenotype and normal Mullerian duct development^11^. Mixed gonadal dysgenesis (mosaicism of 46, XO/46, XY) is a rare case with the presentation of Turner syndrome and prominent virilization. Karyotyping analysis is the only method to diagnose Swyer syndrome and DSD with various sex chromosome characteristics^12^.

There was one subject who had been predicted to have a Y chromosome. Apparently, the karyotyping result was 46, XX. She had been diagnosed with central amenorrhea and Mullerian duct agenesis. Kanadi Sumapraja criteria are assessed within several steps, with the first assessment being to determine the presence of the uterus. The next step is determining the external genitalia and Tanner stage (pubic hair and breast development). Since no uterus existed in phenotypically female external genitalia without developing breast and pubic hair, this subject was classified into category two. It was predicted to have a Y chromosome. The absence of a uterus will exclude the subject from Category 1 (since there was no secondary sexual development) and Category 4B (since the genitalia were not ambiguous). The coincidental finding (central amenorrhea followed by Mullerian duct abnormality) made the Modified Kanadi Sumapraja criteria less accurate.

The sensitivity and specificity of these criteria are reasonable, with 80% sensitivity and 97.96% specificity. The high sensitivity of this criteria, Kanadi Sumapraja criteria is suitable for screening Y chromosome in primary amenorrhea patients because of the high sensitivity of these criteria. These criteria also have a positive predictive ratio of 39.2 and a negative predictive ratio of 0.2. From statistical analysis, the prevalence of primary amenorrhea related to the Y chromosome is up to 16.95%. Thus the positive predictive value is 88.89%. The different prevalence rates will interfere with positive predictive value results. Different geographical areas and specific groups like ambiguous genitalia cause prevalence rate differences. The wide margin of the confidence interval in sensitivity, positive predictive value, and the ratio were most likely due to the number of subjects with a Y chromosome (10 out of 59 subjects).

The development of Kanadi Sumapraja criteria should change the diagnostic investigation workflow of primary amenorrhea cases, especially in rural areas where karyotyping tests are unavailable. Current standardized diagnostic workup for primary amenorrhea starts with evaluation of uterus presence using ultrasound. If the uterus is absent, it is mandatory to perform karyotyping subsequently. With the Kanadi Sumapraja criteria, we could postpone the need for routine karyotyping tests. The karyotyping test would be needed just to reconfirm the suspicion of having XY chromosomes, which are categorized in category 2 or 3.

The strength of this study is the first study to determine the screening tool for the Y chromosome in primary amenorrhea patients. To our knowledge, this is the first study on this topic. The limitation of this study is the unequal number of Y chromosomes detected in the sample. For further development, a wide-scale, multicentred prospective study should be performed for more relevant and accurate data. The laboratory parameters should be included for further studies.

In conclusion, Modified Kanadi Sumapraja criteria could screen the Y chromosome involvement in primary amenorrhea patients with an accuracy of 94.92%, a sensitivity of 80% (49.02–94.33%; [95%-CI]), specificity of 97.96% (89.31–99.64%; [95%-CI]), the positive predictive value of 88.89% (56.5–98.01%; [95%-CI]), and negative predictive value of 96% (86.54–98.9%; [95%-CI]).

## Data Availability

The datasets generated during and/or analysed during the current study are available from the corresponding author upon reasonable request.
